# Histiocytic Sarcoma Secondary to Gastrointestinal Stromal Tumors: A Literature Review

**DOI:** 10.7759/cureus.33055

**Published:** 2022-12-28

**Authors:** Shafi Rehman, Rashid Iqbal, Mahnoor Sukaina, Shameera Shaik Masthan, Amna Bint I Munir, Yagana Iqbal, Muhammad H Qureshi, Ali Husnain, Sana Ghafoor, Bushra Ghafoor, Jai S Nagarajan, Fariyal Pervaiz, Muhammad Haseeb ul Rasool

**Affiliations:** 1 Pathology, Shifa College of Medical Technology, Shifa Tameer-E-Millat University, Islamabad, PAK; 2 Medical Biochemistry, Shifa College of Medical Technology, Shifa Tameer-E-Millat University, Islamabad, PAK; 3 Medicine, Karachi Medical and Dental College, Karachi, PAK; 4 Medicine, University of Louisville, Louisville, USA; 5 Medicine, Fatima Jinnah Medical University, Lahore, PAK; 6 Internal Medicine, Allama Iqbal Medical College, lahore, PAK; 7 Internal Medicine, Mayo Hospital, Lahore, PAK; 8 Medicine, Mayo Hospital, Lahore, PAK; 9 Internal Medicine, Shalimar Clinic, Islamabad, PAK; 10 Research, University of Texas Southwestern Medical Center, Dallas, USA; 11 Medicine, Dera Ghazi Khan Medical College, Dera Ghazi Khan, PAK; 12 Internal Medicine, Nishtar Medical University, Multan, PAK; 13 Medicine, Sri Ramaswamy Memorial (SRM) Medical College Hospital and Research Centre, Chennai, IND; 14 Anatomy, Radiology, Cardiac Catheterization, Shifa College of Medical Technology, Shifa Tameer-E-Millat University, Islamabad, PAK; 15 Medicine, Icahn School of Medicine at Mount Sinai, Queens Hospital Center, New York City, USA

**Keywords:** coexistence, case reports, rare disease, gastrointestinal stromal tumors (gists), histiocytic sarcoma

## Abstract

Histiocytic sarcoma (HS) is a rare tumor that may result from the transdifferentiation of preexisting hematolymphoid neoplasms in a subset of patients. There are instances of correlation or concurrence between HS and a number of cancers, particularly B-cell-associated hematopoietic tumors. Only three cases of HS occurring subsequent to or concurrently with gastrointestinal stromal tumors (GIST) have been recorded. Our main objective was to give an overview of demographics, clinical signs and symptoms, histopathological findings, and immunohistochemical and molecular analysis when HS develops secondary to or concurrently with GIST. A search of PubMed, Google Scholar, and ScienceDirect was undertaken using Medical Subject Headings (MeSH) keywords. According to the findings of our review, there were two males (66.6%) and one female (33.3%). The average age of patients at presentation was 59.6 years. On the immunohistochemistry, three patients were positive for cluster of differentiation (CD) 68 (100%), two patients were positive for CD 163 (67%), one patient was positive for leukocyte common antigen (LCA) (33%), and only one patient was positive for CD 4, CD 10, CD 31, CD 45, human leukocyte antigen (HLA)-DR, lysozyme, and vimentin (33%). On molecular investigation, the gastric mass of only one patient (33.33%) contained a KIT mutation on exon 11. Emperipolesis was observed in one patient (33.33%) on histological examination. Our study provides an important overview of the available literature and gives insight into important diagnostic markers of HS when it occurs secondary to or concurrently with GIST.

## Introduction and background

Histiocytic sarcoma is an extremely rare tumor with an incidence of 0.17 per million individuals [1]. It is characterized by histiocyte-like morphologic and immunophenotypic characteristics [2]. In 2016, the World Health Organization (WHO) classified histiocytic sarcoma among the malignancies of the cell lineage of macrophages and dendritic cells, histiocytoses, myeloid-derived and stromal-derived dendritic cell cancers [3, 4]. Due to its rarity and overlap with numerous mimics like anaplastic large-cell lymphoma and diffuse large B-cell lymphoma, histiocytic sarcoma can be challenging to diagnose [2].

Histiocytic sarcoma often involves extranodal regions, including the digestive tract, soft tissue, lung, and nasal cavity [5]. There have also been reports of lymph nodes [6], cutaneous [7], and brain histiocytic sarcomas [8]. The clinical appearance of histiocytic sarcoma patients varies, ranging from localized disease with a solitary mass to widely disseminated disease [2]. The spleen, lymph nodes, lung, bone marrow, skin, brain, and joints of the limbs are the places where localized histiocytic sarcoma lesions are most frequently discovered [9]. Malignant histiocytosis and disseminated histiocytic sarcoma are multi-system, quickly progressing diseases that simultaneously affect a number of organs, including the spleen, lymph nodes, lung, bone marrow, and skin [9]. In individuals with disseminated disease, the median survival time for patients with histiocytic sarcoma is six months [1]. Patients with localized histiocytic sarcoma may live for years following the initial diagnosis and rigorous clinical management [1,5,6]. Histiocytic sarcoma is treated with surgical excision, chemotherapy, and radiation therapy [2].

Grossly, a fleshy mass with a well-circumscribed or infiltrative border, fluctuating bleeding, or necrosis frequently characterizes the presentation of histiocytic sarcoma [5]. Histologically, a histiocytic sarcoma is made up of sheets of discohesive big polygonal cells with epithelioid to pleomorphic morphology, a lot of eosinophilic to vacuolated or foamy cytoplasm, ovoid to irregularly shaped nuclei, and variable amounts of conspicuous nucleoli. In a small number of cases, focal regions with spindled cells may be visible. A mixed background inflammatory infiltration is frequently noticeable, and there is obvious mitotic activity and tumor necrosis [5,6]. Although hemophagocytosis may exist, it is frequently undetectable due to extensive inflammation [6].

Diagnostic fine-needle aspiration is frequently performed on patients with extranodal histiocytic sarcoma. The cytologic characteristics include lymphoplasmacytic or neutrophilic background with lymphoglandular bodies, and emperipolesis [10]. While cytomorphology alone can be extremely difficult to diagnose histiocytic sarcoma and requires a thorough immunohistochemistry workup, fine-needle aspiration may be a helpful method to detect disease recurrence [10]. At least two of the markers cluster of differentiation (CD) 68, CD 163, CD 4, and lysozyme have to be positive on immunohistochemistry to diagnose histiocytic sarcoma [3].

Gastrointestinal stromal tumors (GIST) are the most common mesenchymal tumor of the gastrointestinal tract and are located in the stomach (56%), followed by small bowel (32%), colorectum (six percent), and esophagus (less than one percent) [11]. The omentum, mesentery, and peritoneum are rarely involved [12]. Approximately 95% of GISTs have CD 117 positivity, 60% to 70% have CD 34 positivity, 30% to 40% have smooth muscle actin positivity, 5% have S100 protein positivity, and 1% to 2% have desmin positivity [13]. Interstitial cells of Cajal (ICC), which are believed to operate as pacemakers in the digestive system, have been identified as the most plausible GIST progenitor cells [11,12]. ICCs exhibit both smooth muscle and neuronal differentiation's immunophenotypic and ultrastructural characteristics [14]. GISTs are thought to develop from CD 34-positive ICC stem cells in the GI tract's wall and differentiate into pacemaker cells [15-17]. Due to the fact that both cell types can express the KIT protein and CD 34, a connection between ICCs and GISTs has been suggested [18].GISTs have remarkable variability in their differentiation pathways and can be roughly classified into four groups: those that differentiate toward smooth muscle cells, those that differentiate toward neural-type cells, those that differentiate toward both smooth muscle and neural-type cells, and tumors that do not differentiate toward either cell type [19]. GIST has morphologically spindle cell development, epithelioid cell growth, and mixed cell growth patterns [20].

Several studies report an association or concurrence of HS with a number of malignancies [21-37].To date, only three cases of HS secondary or concomitant to GIST have been recorded [36-40]. According to our knowledge, this is the first comprehensive review of demographics, clinical signs and symptoms, histopathological findings, and immunohistochemical and molecular analysis of case reports when HS develops secondary to or concurrently with GIST.

## Review

Methodology

To investigate cases of histiocytic sarcoma occurring secondary or concurrently to GIST, we used PubMed, Google Scholar, and ScienceDirect. We collected all relevant reports electronically by entering the necessary keywords. The search cutoff date for databases was July 10, 2022. We implemented a Boolean approach using Medical Subject Heading (MeSH) keywords. When establishing the inclusion/exclusion criteria listed below, article titles and abstracts were evaluated. In this review, Preferred Reporting Items for Systematic Reviews and Meta-Analyses (PRISMA) standards for 2020 were followed [41].

Inclusion and exclusion criteria

We included only English-language case reports and conference abstracts with unrestricted access to full-text reports and with only human subjects. We excluded all other study designs. MeSH keywords searched in PubMed are summarized in Table 1.

**Table 1:** MeSH search strategy of PubMed MeSH - Medical Subject Headings,

Search strategy	PubMed results
(#1) Histiocytic Sarcoma OR Histiocytic Sarcomas OR True Malignant Histiocytoses OR True Histiocytic Lymphoma OR True Histiocytic Lymphomas OR Malignant Histiocytoses OR Malignant Histiocytosis OR ("Histiocytic Sarcoma/anatomy and histology"(MeSH) OR "Histiocytic Sarcoma/classification"(MeSH) OR "Histiocytic Sarcoma/diagnosis"(MeSH) OR "Histiocytic Sarcoma/diagnostic imaging"(MeSH) OR "Histiocytic Sarcoma/epidemiology"(MeSH) OR "Histiocytic Sarcoma/etiology"(MeSH) OR "Histiocytic Sarcoma/genetics"(MeSH) OR "Histiocytic Sarcoma/immunology"(MeSH) OR "Histiocytic Sarcoma/pathology"(MeSH))	3169 studies
(#2) Gastrointestinal Stromal Tumor OR Gastrointestinal Stromal Tumors OR Gastrointestinal Stromal Neoplasm OR Gastrointestinal Stromal Neoplasms OR ("Gastrointestinal Stromal Tumors/anatomy and histology"(MeSH) OR "Gastrointestinal Stromal Tumors/classification"(MeSH) OR "Gastrointestinal Stromal Tumors/complications"(MeSH) OR "Gastrointestinal Stromal Tumors/cytology"(MeSH) OR "Gastrointestinal Stromal Tumors/diagnosis"(MeSH) OR "Gastrointestinal Stromal Tumors/diagnostic imaging"(MeSH) OR "Gastrointestinal Stromal Tumors/epidemiology"(MeSH) OR "Gastrointestinal Stromal Tumors/etiology"(MeSH) OR "Gastrointestinal Stromal Tumors/genetics"(MeSH) OR "Gastrointestinal Stromal Tumors/immunology"(MeSH) OR "Gastrointestinal Stromal Tumors/pathology"(MeSH) OR "Gastrointestinal Stromal Tumors/secondary"(MeSH))	12851 studies
(#1) AND (#2)	3 studies

The search strategy in ScienceDirect and Google Scholar is summarized in Table 2.

**Table 2:** Search strategy for ScienceDirect and Google Scholar

	Keywords	Results
ScienceDirect	(Histiocytic Sarcoma OR Histiocytic Sarcomas) AND (Gastrointestinal Stromal Tumor OR Gastrointestinal Stromal Tumors OR Gastrointestinal Stromal Neoplasm OR Gastrointestinal Stromal Neoplasms)	5 results
Google Scholar	Histiocytic Sarcoma AND Gastrointestinal Stromal Tumors	200 results

Results

**Figure 1:**
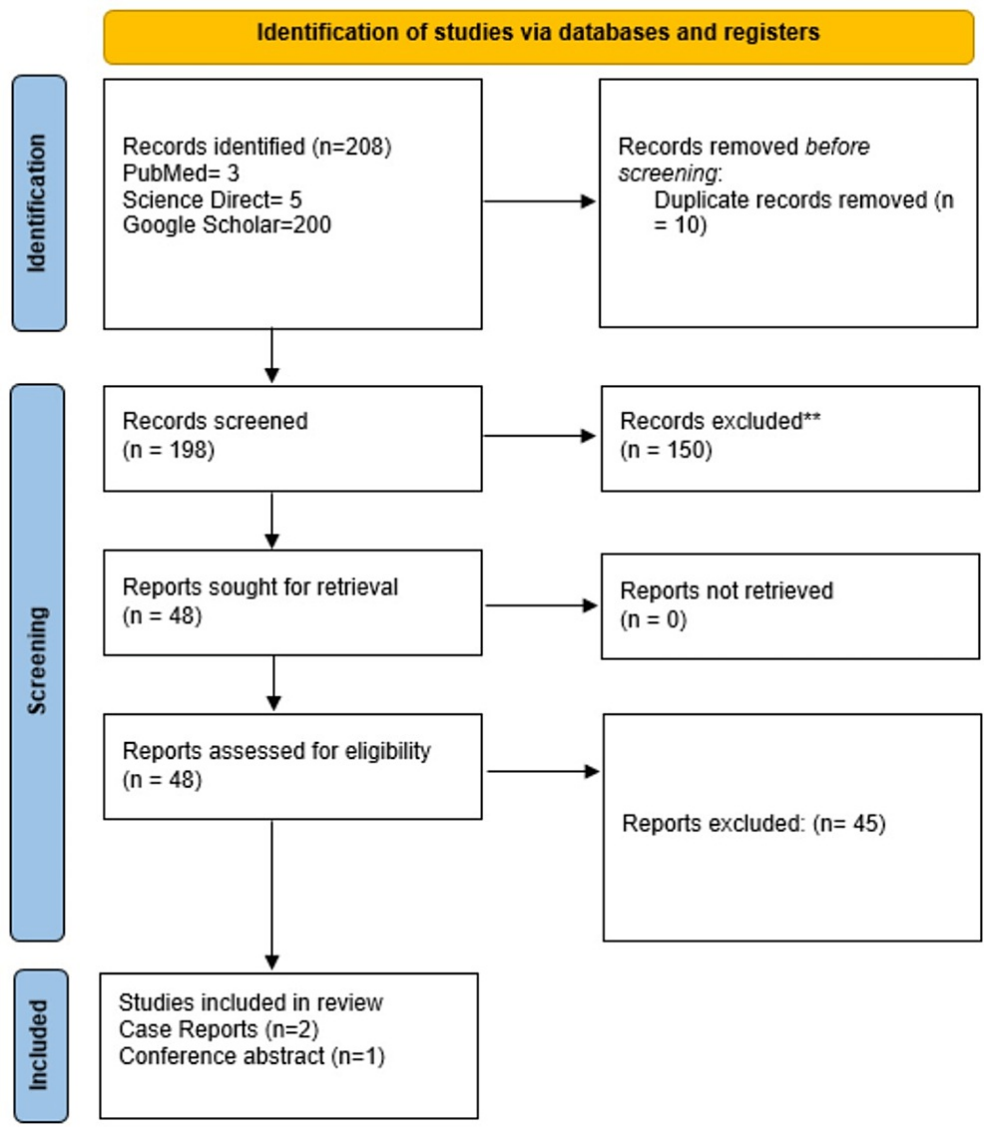
PRISMA 2020 flowchart PRISMA - Preferred Reporting Items for Systematic Reviews and Meta-Analyses

This review's search approach includes the aforementioned databases and generated 208 articles, of which 10 duplicates were eliminated using Zotero. A total of 198 records were reviewed, and 150 were discarded based on the inclusion/exclusion and relevance criteria. The final screening yielded 48 reports for quality and eligibility evaluation. Lastly, one conference abstract and two case reports were included in this review. A quality assessment tool using the Joanna Briggs Institute (JBI) Critical Appraisal Checklist for Case Reports was performed. The PRISMA flowchart is illustrated in Figure 1.

In our review, there were two males (66.6%) and one female (33.3%) as per gender distribution. The mean age of presentation was 59.6 years. On the immunohistochemistry panel, all three patients were positive for CD 68 (100%), two patients were positive for CD 163 (67%), one patient was positive for leukocyte common antigen (LCA; 33%), and only one patient was positive for CD 4, CD 10, CD 31, CD 45, HLA-DR, lysozyme, and vimentin (33%). Two patients (66.6%) were positive for both CD 68 and CD 163, and one patient (33.3%) was positive for CD 68 and LCA. On molecular analysis, the gastric mass was positive for a KIT mutation on exon 11 in one patient (33.33%). In two patients (67%), there was no BRAF mutation, and molecular analysis was not done in one patient (33.3%). On histopathological findings, emperipolesis was seen in one patient (33.33%). The results of our review are summarized in Table 2 and Table 3. 

**Table 3:** Summary of case reports GIST - gastrointestinal stromal tumor, TCR - T-cell receptor

Author	Gender	Age	Past medical history and treatment	Presenting symptom	Imaging findings	Gross examination	Histopathological findings	Molecular analysis
Tong et al. (2021) [38]	Female	68 years	Six years ago, GIST was in remission with imatinib and partial gastrectomy	Pain in the abdomen and chronic constipation	CT scan: gastric mass in the upper left quadrant of the abdomen, originating from the posterior portion of the gastric body next to the greater curvature. It was 13.1 × 11.4 ×10.7 cm in size. Endosonography: a round intramural lesion in the body of the stomach that arises from the muscularis propria and involves the submucosa layer.	Grossly, a mass lesion of 9.5 centimeters was observed between muscularis propria and serosa. The lesion consisted of a major hollow (6.0 cm) bordered by two little discrete nodules (1.0 cm and 0.6 cm). The lesion was composed of fibrotic stroma (80%of the bulk), with postchemotherapy effects.	Both small nodules were composed of big atypical epithelioid cells with rich eosinophilic, coarsely granulated cytoplasm and spherical nuclei with conspicuous nucleoli. Mitotic figures climbed dramatically (approximately seven per 10 high power field). There were little reactive lymphocytes in the background.	Molecular examination did not reveal any BRAF mutations.
Gangoli et al. (2016) [39]	Male	54 years	Stomach GIST previously treated with surgical excision and Imatinib therapy two years prior	Pain in abdomen	Imaging revealed a lesion in the small intestine. No further lesions were found in the lymph nodes.	Grossly, a yellowish-brown, spherical, mucoid mass was observed in the small intestine section that had been removed. The majority of the mass consisted of submucosa and muscularis propria. At times, it was observed to be close to the serosal surface. Necrosis areas were identified. Although the intestinal lumen looked to be constricted, the mucosa was unremarkable.	The tumour consisted of diffuse sheets of pleomorphic, round to oval cells with rich eosinophilic cytoplasm including small granules, as observed under the microscope. The cells possessed vesicular nuclear chromatin with conspicuous nucleoli, a high mitotic activity, and occasional aberrant mitosis. The defining characteristic was widespread emperipolesis. A polymorphic population consisting of neutrophils, lymphocytes, and plasma cells was observed in the background. There were also several multinucleated giant cells present.	No molecular investigation for the identification of immunoglobulin heavy chain and gene rearrangement of TCR Gamma chain was conducted.
Obiorah et al. (2018) [40]	Male	57 years	N/A	N/A	N/A	N/A	N/A	Positive for a KIT mutation on exon 11 and negative for a BRAF v600 mutation on the gastric mass.

**Table 4:** Immunohistochemistry findings CD - cluster of differentiation, HLA - human leukocyte antigen, ALK-1 - anaplastic lymphoma kinase-1, AE1/AE3 - anti-cytokeratin monoclonal antibodies, CAM5.2 - cytokeratin, DOG-1 - discovered on gastrointestinal stromal tumor-1, EMA - epithelial membrane antigen, HepaPar-1 - hepatocyte paraffin-1, HMB-45 - human melanoma black, MART-1 - melanoma antigen recognized by T-cells, MPO - myeloperoxidase, S-100 - soluble in 100%, SMA - smooth muscle actin, Tdt - terminal deoxynucleotidyl transferase, WT-1 - Wilms tumor-1, Ki-67 - marker of proliferation, LCA - leukocyte common antigen, Pan-CK - pan cytokeratin

Marker	Case 1	Case 2	Case 3
CD4	+ve	-ve	-ve
CD 10	+ve	-ve	-ve
CD 31	+ve	-ve	-ve
CD 45	+ve	-ve	-ve
CD 68	+ve	+ve	+ve
CD 163	+ve	-ve	+ve
HLA-DR	+ve	-ve	-ve
Lysozyme	+ve	-ve	-ve
Vimentin	+ve	-ve	-ve
ALK-1	-ve	-ve	-ve
AE1/AE3	-ve	-ve	-ve
Calretinin	-ve	-ve	-ve
CAM5.2	-ve	-ve	-ve
CD 1a	-ve	-ve	-ve
CD 3	-ve	-ve	-ve
CD 15	-ve	-ve	-ve
CD 20	-ve	-ve	-ve
CD 21	-ve	-ve	-ve
CD 30	-ve	-ve	-ve
CD 34	-ve	-ve	+ve
CD 35	-ve	-ve	-ve
CD 43	-ve	-ve	-ve
CD 117 (c-kit)	-ve	-ve	+ve
CD 123	-ve	-ve	-ve
Chromogranin	-ve	-ve	-ve
Desmin	-ve	-ve	-ve
DOG-1	-ve	-ve	-ve
EMA	-ve	-ve	-ve
HepaPar-1	-ve	-ve	-ve
HMB-45	-ve	-ve	-ve
MART-1	-ve	-ve	-ve
MPO	-ve	-ve	-ve
S100	-ve	-ve	-ve
SMA	-ve	-ve	-ve
Synaptophysin	-ve	-ve	-ve
TdT	-ve	-ve	-ve
WT-1	-ve	-ve	-ve
Ki-67	20-25%	N/A	N/A
LCA	-ve	+ve	-ve
Pan-CK	-ve	-ve	-ve

Review of literature

Transdifferentiation is the process of changing a cell type from one tissue or organ into a cell type from a different tissue or organ directly without going through any intermediate cell lineage. It is also called lineage reprogramming [42]. According to previous publications, HS has been linked to or co-occurred with a number of cancers [21-37]. These papers have offered both direct and indirect proof that the two otherwise morphologically and immunohistochemically distinct neoplasms could transdifferentiate. Only three cases of HS and GIST have been documented thus far [38-40]. Two years following therapy, a patient with previously treated GIST in the stomach was found to have one case of HS in the intestine [39]. In another report, synchronous HS and GIST in the abdomen were described [40]. According to Tong et al., a 68-year-old woman who underwent a partial gastrectomy and imatinib-based treatment for stomach GIST developed HS. Two really interesting happenings in this context were seen. First, although having different immunophenotypes and cellular sizes, the epithelioid appearance of GIST and HS tumor cells was comparable. Second, the two HS-positive nodules, which were the location of the prior GIST biopsy, were discovered on the inner surface of the mass cavity with stromal fibrosis. This may have shown that the HS appeared concurrently with the GIST or afterward [38]. It has been noted that there might be a delay of up to seventeen years between the beginning of lymphoma and HS [43,44]. In the case study given by Tong et al., the patient received imatinib therapy two years prior to the mass's recurrence and once after it. Chronic myeloid leukemia can be effectively treated with imatinib therapy (CML). Imatinib discontinuation trials typically have two distinct outcomes, with one group relapsing and the other remaining disease-free during the follow-up period [45]. According to Tong et al. [38], CML patients treated with imatinib lead a rise of leukemic clones, which have different growth and differentiation properties compared to the predominant clone at the start of therapy; therefore, in response to tissue damage, transdifferentiation across cell types can happen both naturally and synthetically. It has been demonstrated that inhibiting KIT signaling results in the transdifferentiation of interstitial cells of Cajal into smooth muscle cells [46]. Tong et al., therefore, hypothesized that perhaps Cajal interstitial cells' interaction with imatinib and other intrinsic host factors, like elements of the immune system and the tumor microenvironment, leads to transdifferentiation toward HS [38]; however, it's too early to presume this, and we need firm evidence based on large multi-center cohort studies to arrive at this interesting conclusion.

Histiocytic sarcoma is characterized by a broad age distribution and a significant predominance of males [2]. This correlates to the findings of our review as there were two men (66.6%) and one female (33.3%), with an average age of 59.6 years. Histiocytic sarcoma frequently affects extranodal locations, such as the digestive tract, soft tissue, lung, and nasal cavity [5]. The most frequent mode of presentation involves the intestine and is characterized by abdominal pain and intestinal obstruction [47]. In accordance with our findings, abdominal discomfort was the most prevalent complaint at the presentation. Two out of three patients first reported abdominal pain in our review. Generally, HS is polypoidal and contains malignant histiocytes with rich eosinophilic cytoplasm and pleomorphic, vesicular nuclei with conspicuous nucleoli, as seen under a microscope. Necrosis is frequently observed. These tumors are characterized by widespread and pronounced emperipolesis. Emperipolesis differs from phagocytosis in that it involves the structurally and physiologically viable engulfment of a living cell by a histiocyte or platelet. In addition to HS, it is pathognomonic of histiocytic illnesses, such as Rosai-Dorfman disease, and hemato-lymphoid disorders, such as myeloid leukemias and lymphomas [48]. Only one patient's histopathological findings showed emperipolesis in our review.

In order to confirm histiocytic differentiation with positive markers and to rule out morphologically similar entities with negative markers, immunohistochemistry is crucial for the diagnosis of histiocytic sarcoma. A small percentage of instances of histiocytic sarcomas additionally express CD 31, CD 4 (cytoplasmic), and CD 45RO [5,6,10,21]. The majority of these tumors express the positive tumor markers CD 68, CD 163, and PU.1. This agrees with what we found because three patients in our review tested positive for CD 68 (100%) and two patients for CD 163 (67%), respectively. The expression of at least two of the following markers-CD 68, CD 163, CD 4, and lysozyme-has been suggested as a diagnostic criterion for histiocytic sarcoma [3]. Although a-1-antitrypsin, lysozyme, and CD 68, the clones KP-1 and PGM-1, were used in earlier studies, each of these markers is not only exclusive to the histiocytic lineage but can also be expressed in other neoplasms, such as melanoma [6]. There has been a rise in the use of CD 163 and PU.1 in the diagnosis of histiocytic sarcoma and other histiocytoses. When compared to CD 68, CD 163 is more accurate in identifying the histiocytic lineage and making the diagnosis of histiocytic sarcoma [6,25,49]. In spite of the significant background inflammation, the transcription factor PU.1, a macrophage lineage marker, can be particularly useful for identifying and validating the nuclear immunoreactivity in tumor cells [10,25].

Patients with follicular lymphoma and histiocytic sarcoma have both been identified as having immunoglobulin H (IgH)-BCL2 fusions [25,26]. In both mantle cell lymphoma and histiocytic sarcoma, one patient was shown to have a fusion of cyclin D1 and IgH [28]. The BRAF mutant V600E has been found in both histiocytic sarcoma and hairy cell leukemia in one patient with concurrent diseases, further indicating a common genetic ancestor [29]. According to recent studies, certain histiocytic sarcomas include BRAF alterations, including V600E and non-V600E variants [29,44,46]. Additionally, it has been discovered that three cases of histiocytic sarcoma have recurrent mutations in the KMT2D gene, which is associated with epigenetic regulation and frequently altered in follicular lymphomas [19]. A lot of neoplasms, both hematological and nonhematopoietic, have been shown to carry the BRAF V600 E point mutation, which turns on the BRAF pre-oncogene. Four to thirteen percent of patients with KIT/PDGFRA wild-type GIST carry the BRAF V600 E mutation, despite the fact that KIT and PDG are the most common mutations in GIST [50, 51]. The BRAF V600 E mutation has also been discovered in some HS cases [30,52,53]. In one study, the BRAF V600 E mutant was discovered in five of eight HS subjects [52]. Historical and present specimens were examined for BRAF mutations to see if the BRAF V600 E mutant acts as a driver mutation favoring malignant transdifferentiation from GIST to HS [52]. According to the results of our systematic review, only one patient's stomach mass had a KIT mutation on exon 11, and there was no BRAF mutation.

Using targeted next-generation sequencing, Hornick's team has discovered various activating mutations in the RAS-MAPK, PI3K-AKT-MTOR, and CD KN2A tumor-suppressor gene in the majority of histiocytic sarcomas [54]. Furthermore, a subset of these mutation-carrying patients shares striking genetic similarities with B-cell lymphomas [54]. Whether these mutations coexist in our GIST and HS patients is unknown. In the future, the targeted next-generation sequencing method will be a crucial tool for examining these alterations and illuminating the most likely pathway of GIST to HS transdifferentiation.

Limitations

The biggest limitation of our literature review was the presence of only two case reports and one conference abstract with a sample size of three, due to which we can't draw any significant conclusion. The ethnicity of patients was missing in all reports. There were no significant details on the management and follow-up of the patients. Therefore, future cases should include detailed parameters to add value to the literature.

## Conclusions

Our review summarizes available data when HS occurs secondary to or concurrently with GIST. All three patients had confirmed HS in our review and were positive for CD 68. Only two patients were positive for CD 163. Two patients had HS with GIST treated previously, and one patient had concomitant GIST with positive CD 68, CD 117, and CD 34. The most common clinical presentation was abdominal pain. Only one patient had emperipolesis on histopathological examination. Our knowledge of the exact relationship between HS and GIST is limited given less sample size and needs to be supplemented by a large sample size; therefore, rare cases like these need to be reported. Our review is important in the sense that it provides an overview of the current available literature and gives insight into important diagnostic markers when HS occurs secondary to or concurrently with GIST.
